# Severe hyperlactatemia in unselected surgical patients: retrospective analysis of prognostic outcome factors

**DOI:** 10.1186/s12893-022-01729-2

**Published:** 2022-08-11

**Authors:** Julia Spiegelberg, Ann-Kathrin Lederer, Sibylla Claus, Mira Runkel, Stefan Utzolino, Stefan Fichtner-Feigl, Lampros Kousoulas

**Affiliations:** 1 Department of General and Visceral Surgery, Medical Center-University of Freiburg, Hugstetter Straße 55, 79106 Freiburg, Germany; 2 Center for Complementary Medicine, Department of Internal Medicine II, Medical Center-University of Freiburg, Faculty of Medicine, University of Freiburg, Freiburg, Germany

**Keywords:** Lactate, Lactic acidosis, Sepsis, Shock, Outcome, Surgical therapy

## Abstract

**Background:**

Etiology of hyperlactatemia in ICU patients is heterogeneous—septic, cardiogenic or hemorrhagic shock seem to be predominant reasons. Multiple studies show hyperlactatemia as an independent predictor for ICU mortality. Only limited data exists about the etiology of hyperlactatemia and lactate clearance and their influence on mortality. The goal of this single-center retrospective study, was to evaluate the effect of severe hyperlactatemia and reduced lactate clearance rate on the outcome of unselected ICU surgical patients.

**Methods:**

Overall, 239 surgical patients with severe hyperlactatemia (> 10 mmol/L) who were treated in the surgical ICU at the University Medical Center Freiburg between June 2011 and August 2017, were included in this study. The cause of the hyperlactatemia as well as the postoperative course and the patient morbidity and mortality were retrospectively analyzed. Lactate clearance was calculated by comparing lactate level 12 h after first measurement of > 10 mmol/L.

**Results:**

The overall mortality rate in our cohort was 82.4%. Severe hyperlactatemia was associated with death in the ICU (p < 0.001). The main etiologic factor was sepsis (51.9%), followed by mesenteric ischemia (15.1%), hemorrhagic shock (13.8%) and liver failure (9.6%). Higher lactate levels at ICU admission were associated with increased mortality (p < 0.001). Lactate clearance after 12 h was found to predict ICU mortality (ANOVA p < 0.001) with an overall clearance of under 50% within 12 h. The median percentage of clearance was 60.3% within 12 h for the survivor and 29.1% for the non-survivor group (p < 0.001).

**Conclusion:**

Lactate levels appropriately reflect disease severity and are associated with short-term mortality in critically ill patients. The main etiologic factor for surgical patients is sepsis. When elevated lactate levels persist more than 12 h, survival chances are low and the benefit of continued maximum therapy should be evaluated.

## Background

Hyperlactatemia is defined as lactate level > 2 mmol/L and is commonly seen in critically ill patients [1]. Hyperlactatemia results from the accumulation of lactate and protons in human fluids and tissues and is often associated with poor clinical outcomes. When aerobic glycolysis is impaired, cells increase their glucose utilization by anaerobic glycolysis. This process is less efficient in generating triphosphates and additionally produces pyruvate. This undergoes redox-coupled interconversion catalyzed by the enzyme lactate dehydrogenase, ultimately resulting in lactate [2]. Hyperlactatemia occurs when lactate production outruns lactate consumption. Additionally, protons equivalent to the number of excess lactate ions are synthetized regardless of the prevailing acid–base status. Approximately 70% of lactate clearance occurs in the liver. Coexisting acidemia contributes to decreased lactate clearance by the liver. Therefore the severity of acidemia seems to be a better predictor of cellular dysfunction and clinical outcomes than hyperlactatemia [3].

In general, hyperlactatemia is caused by tissue hypoxemia due to an imbalance between oxygen supply and demand. This tissue hypoxemia is attributed by impairment of oxygen supply or different forms of shock such as hypovolemic, hemorrhagic, cardiogenic or obstructive shock. Nevertheless, hyperlactatemia can also occur under aerobic conditions. During a state of shock, lactate production is dependent on the stimulation of β_2-_ muscle receptors, thus largely independent of tissue hypoxia [4]. Lactate levels and their trend may be reliable markers of illness severity and mortality [5, 6]. Only limited data exists about the etiology of lactatemia in sepsis. Wong et al. included lactate in a multi biomarker-based outcome risk model for patients with septic shock [7]. The metabolism of lactate in critically ill patients is described as being associated with cellular inflammatory response [8]. Thus, in summary, both lactate production and clearance appear to play a critical pathophysiological role and to be crucial for outcome and survival.

Hyperlactatemia is considered as a clinical marker of critical illness severity. The association between elevated lactate levels and poor outcome of ICU patients is reported in multiple studies [9]. Effective lactate clearance seems to be associated with improved outcome [10]. Recently, Haas et al. retrospectively analyzed patients with plasma lactate levels > 10 mmol/L [11], and found an overall mortality of 78.2% in these patients, whereas mortality rate of all patients in ICU were only 9.8%. The main etiology for severe hyperlactatemia was septic shock (34.0%), cardiogenic shock (19.3%) and cardiopulmonary resuscitation (13.8%) [11]. Nevertheless, the etiology of hyperlactatemia and lactate clearance rates showed heterogeneity emphasizing the necessity of further research. We therefore analyzed severe hyperlactatemia in unselected surgical patients from the surgical intensive care unit of the University Medical Center Freiburg and evaluated prognostic outcome factors. We hypothesized that severe hyperlactatemia (> 10 mmol/L) and a reduced lactate clearance are associated with a poor outcome of surgical patients.

## Methods

This cohort study retrospectively evaluated the outcome of 239 consecutive surgical patients with serum lactate levels > 10 mmol/L, who were treated at the Medical Center of the University Freiburg between June 2011 and August 2017. The study was approved by the medical Ethics Committee of the University of Freiburg (EK-FR 372/17).

Patients were eligible for inclusion if serum lactate concentration > 10 mmol/L was documented in a venous or arterial blood sample on at least one occasion. Only patients being treated in surgical ICU were considered. All patients underwent surgery or received another therapeutic intervention such as endoscopic procedures (gastroscopic clipping, endoscopic retrograde cholangiography), therapeutic interventional angiography or comparable endovascular interventions.

No pre-analytical sample size calculation was performed. Electronic data acquisition of blood gas analysis results in our medical department exists since June 2011. Therefore, due to feasibility reasons, a fixed period between June 2011 and August 2017 was chosen during which all above-mentioned surgical patients with severe hyperlactatemia were included to data analysis.

Lactate concentrations were measured during ICU stay in every patient and recorded by an automated data management system. Blood samples were collected in heparinized blood gas syringes and measured with a blood gas analyzer (ABL800 FLEX © Radiometer; Radiometer, Copenhagen, Denmark). All laboratory maintenance was reviewed regularly and tested according to national German laws and manufacturer’s specification.

Clinical data and patient demographics were extracted from our patient data management system (KIS Freiburg PROMetheus). ICU admission date, date of death (in or outside hospital), cause of hyperlactatemia, sex, age, organic failure / replacement therapies, operations or interventions, hospital admission lactate level as well as peak lactate level and 12 h lactate level were defined as target variables. Patients were grouped into different categories related to the etiology of hyperlactatemia [11]: (1) sepsis (including septic shock), (2) cardiogenic shock, (3) postoperative cardio surgical patients, (4) cardiopulmonary resuscitation, (5) hemorrhagic shock, (6) liver failure, (7) mesenteric ischemia, (8) seizure, (9) other reasons not captured by the previous categories.

SPSS 27 for Windows™ was used for statistical analysis (SPSS, Chicago, IL, USA). Categorical variables are presented as frequency and percentage of level. Quantitative variables are shown as the mean ± standard deviation (SD) of distribution. Lactate clearance was calculated by comparing blood lactate concentration 12 h after first measurement of lactate > 10 mmol/L. Categorical variables were put in absolute and relative frequencies; differences were evaluated by Chi-Square or Fisher’s exact test as appropriate. Quantitative values were expressed as medians with range and differences were measured using the Mann–Whitney-U test. Multivariate analysis was performed through forward logistic regression model, with relative risk and a 95% confidence interval. The Kaplan–Meier method was used to evaluate survival, with a log-rank test for the comparison of subgroups. We used the Area under the Curve (AUC) of the Receiver Operating Characteristic (ROC) curve to prognosticate the predictive power of different factors predicting ICU mortality. Youden Index was used to find the optimal cut-off value, which leads to the highest sensitivity and specificity. A p-value < 0.05 was considered statistically significant.

## Results

Between June 2011 and August 2017, a total of 14.973 patients were treated in the surgical intensive care unit at the University Medical Center Freiburg. Of these, 242 patients with lactate level > 10 mmol/L during ICU stay were eligible for study inclusion. Three patients had to be excluded due to implausibility of the recorded lactate measurements. Therefore, data from 239 patients were available for retrospective evaluation. Of 239 included patients, 18 (7.5%) were admitted after elective surgery or intervention and 221 (92.5%) were emergency ICU admissions due to variable causes. We summarized demographic data as well as maximum lactate level, mean hospitalization days, ICU days, ventilation percentage, renal replacement therapy and etiology of hyperlactatemia in Table 1.

**Table 1:** Demographic data, complications and lactate etiology

Parameter	Total	Survivors	Non-survivors	p^a^
	n = 239	n = 42 (17.6%)	n = 197 (82.4%)	
Mean age (years) ± SD	67.6 ± 12.3	64.1 ± 13.5	68.3 ± 12.0	< 0.001
Gender				
Male	144 (60.3%)	23 (9.6%)	121 (50.6%)	0.264
Female	95 (39.7%)	19 (7.9%)	76 (31.8%)	
Mean duration of ICU stay (days)	10.1 ± 13.8	14.4 ± 13.4	9.2 ± 13.7	0.025*
Hospitalization days (days)	20.6 ± 23.7	37.2 ± 25.8	17.0 ± 21.7	0.000*
Ventilation [n (percentage)]	208 (87.0%)	30 (12.6%)	178 (74.5%)	0.004*
Renal replacement [n (percentage)]	72 (30.1%)	11 (4.6%)	61 (25.5%)	0.340
Maximum lactate (mmol/L)	17.0 ± 5.8	13.9 ± 4.4	17.8 ± 5.8	0.001*
Lactate etiology*				
Sepsis	124 (51.9%)	14 (11.3%)	110 (88.7%)	
Mesenteric ischemia	36 (15.1%)	5 (13.9%)	31 (86.1%)	
Hemorrhagic shock	33 (13.8%)	8 (24.2%)	25 (75.8%)	
Liver failure	23 (9.6%)	7 (30.4%)	16 (69.6%)	
Cardiopulmonary resuscitation	12 (5.0%)	2 (16.7%)	10 (83.3%)	
Cardiogenic shock	6 (2.5%)	2 (33.3%)	4 (66.7%)	
Seizure	2 (0.8%)	1 (50%)	1 (50%)	
Other reasons	3 (1.3%)	3 (100%)	0 (0%)	

Mann–Whitney-U-Test

^a^For lactate etiology/ Fisher’s exact Test for other parameters

* Significant difference between groups regardings Fisher's exact test

In this cohort mortality was 82.4% (n = 197). There was a significant difference in age between ‘survivors’ and ‘non-survivors’ (64 years vs. 68 years, respectively p < 0.001). There was no statistical difference in gender distribution across the two groups. Non-survivors were more likely to be ventilated (p = 0.004), however the need for renal replacement therapy showed no significant difference between the two groups. (p = 0.340).

In the survivor group the mean length of ICU stay was 14.4 ± 13.4 days compared to 9.2 ± 13.7 days for the non-survivor group.

Peak lactate level was significantly higher in the non-survivor group (17.8 ± 5.8 mmol/L) compared to the survivor group (13.9 ± 4.4 mmol/L, p = 0.001).

### Etiology and course of hyperlactatemia

**Fig. 1:**
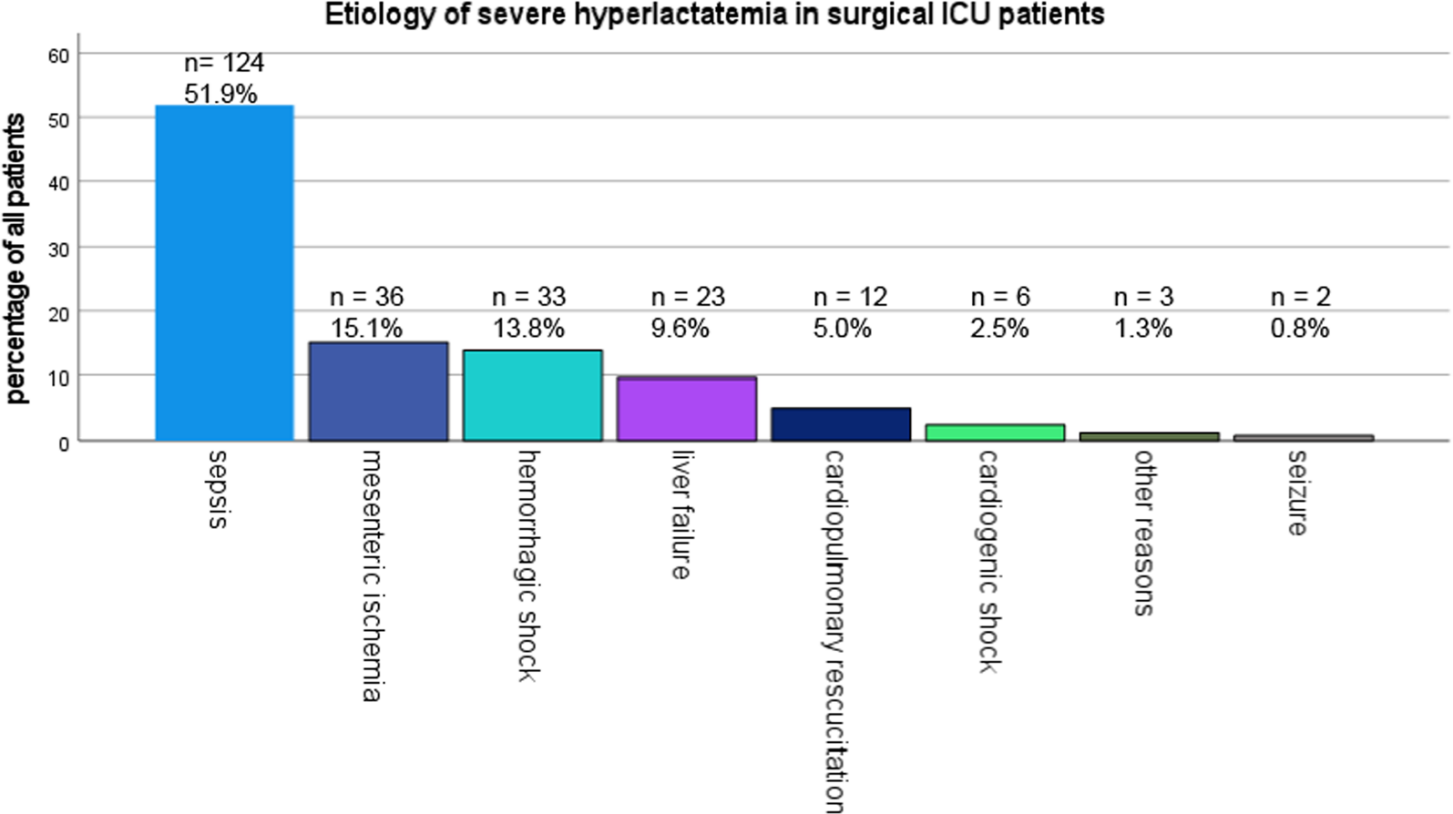
Etiology of hyperlactatemia

The distribution of patients across different groups of etiology of hyperlactatemia is shown in Fig. 1. In 24 cases, the etiology was considered to be multifactorial and the leading cause was determined according to patients’ medical history and current consecutive symptom order.

Mortality rate in this cohort was 82.4% (n = 197). In numerous cases a precise determination of the cause of death was not possible. However, given the frequency of sepsis and septic shock (51.9%), septic multi-organ failure is likely to be the leading cause. Other causes of death included circulatory failure, ARDS and therapy limitation due to, for example, irreversible complete bowel ischemia. ICU mortality in the subgroups was 89% due to sepsis, 86% due to mesenteric ischemia, 76% due to hemorrhagic shock, 70% due to liver failure, 83% due to cardiopulmonary resuscitation, 67% due to cardiogenic shock, 50% due to seizure and 0% due to other reasons. For 72 patients requiring renal replacement therapy, ICU mortality was 85%. Mortality for patients with persisting hyperlactatemia for > 12 h was 87.7% (n = 192, (p < 0.001).

**Fig. 2:**
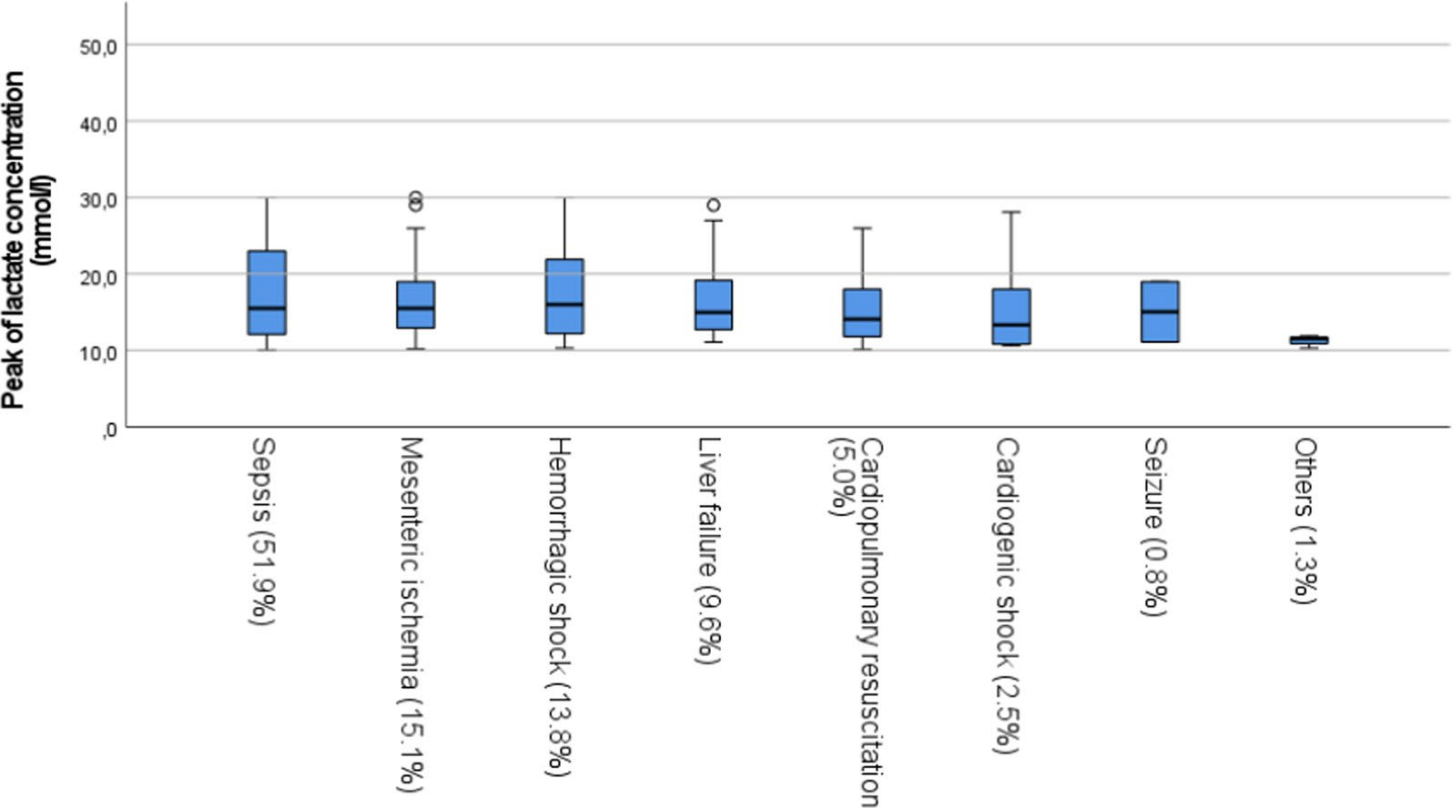
Distribution of measured peak lactate concentration (mmol/L) by subgroups

Median lactate concentration ranged from 11.2 ± 0.8 mmol/L to 17.6 ± 6.0 mmol/L. The distribution of maximum lactate levels is shown in Fig. 2.

Mean lactate concentration was 13.9 ± 4.4 mmol/L in the survivor group and 17.8 ± 5.8 mmol/L in the non-survivor group. In patients with lactate concentration < 10 mmol/L at the time of ICU admission survival was 21.5 ± 2.0 days compared to patients with lactate concentration of > 10 mmol/L with 7.9 ± 1.3 days. We calculated 12.38 mmol/L as peak lactate cut-off with the highest sensitivity for prediction of mortality (Youden Index).

**Fig. 3:**
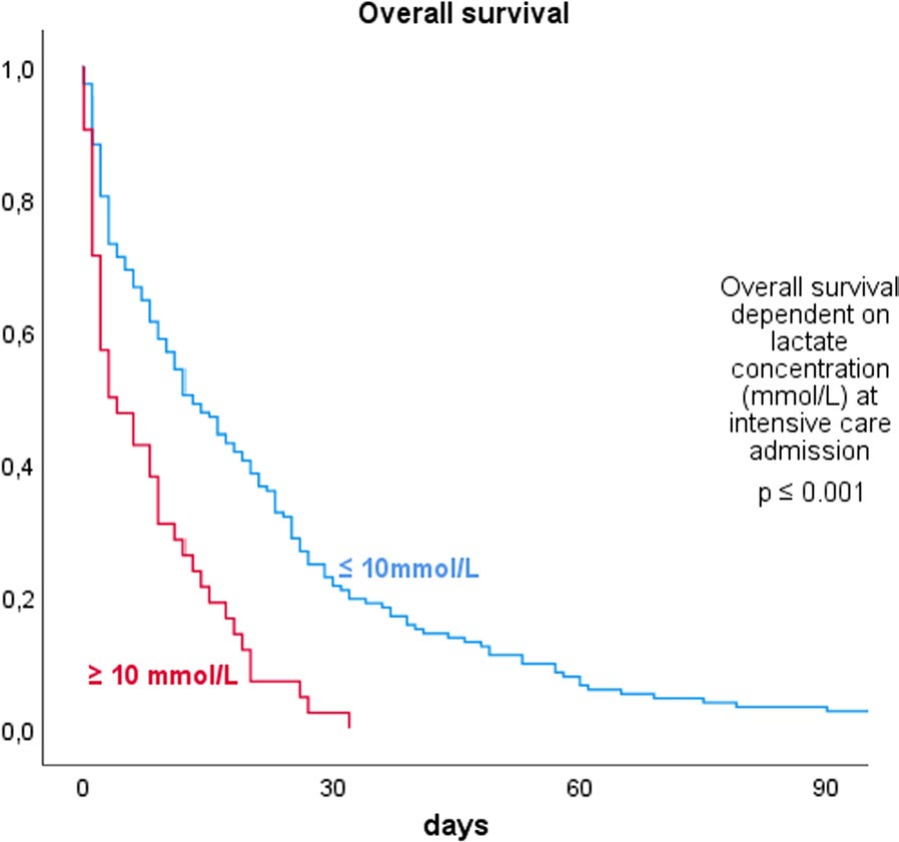
Kaplan-Maier 90 days overall survival depending on blood lactate concentration at ICU admission

When performing Kaplan-Maier analysis, we found blood lactate concentration at ICU admission to significantly influence overall survival (Fig. 3). Patients whose admission lactate concentration was already above 10 mmol/L show a significantly reduced chance of survival in the 90-day overall survival compared to patients with lactate levels < 10 mmol/L, according to log rank analysis (p < 0.001).

**Fig. 4:**
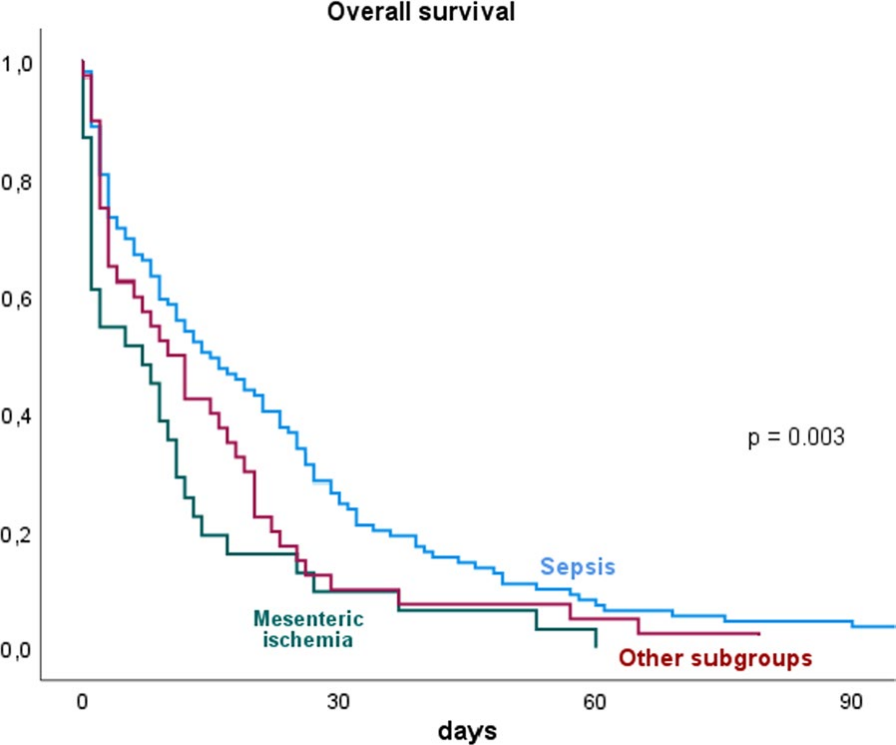
Kaplan-Maier 90 days overall survival in patients with severe hyperlactatemia depending on different etiologic factors (log rank analysis)

Patients with mesenteric ischemia has significantly lower overall survival compared to other subgroups (p = 0.003). Patients with sepsis and septic shock seem to have a better outcome when compared to all other subgroups (Fig. 4).

### Lactate clearance

Data of lactate clearance within 12 h was available for 90 patients (40 survivors, 50 non-survivors). The median percentage of clearance (percentage of reduction after 12 h compared to the first measured value of lactate above 10 mmol/L) was 60.3% within 12 h for the survivor group and 29.1% for the non-survivor group (p < 0.001). There was no statistical difference of 12 h lactate clearance between etiologic subgroups (p = 0.243). Calculation of ROC analysis revealed a cut-off value for 12 h lactate clearance of 29.4% for the prediction of ICU mortality.

Lactate clearance was significantly slower when aspartate aminotransferase (p = 0.013) as well as alanine aminotransferase were increased (p = 0.017). The mean 12 h lactate clearance in each subgroup was 37.7% ± 40.9 for sepsis, 72.6% ± 0 for cardiogenic shock 55.0 ± 50.1% for cardiopulmonary resuscitation, 43.0 ± 39.7% for hemorrhagic shock, 30.5 ± 56.6% for liver failure, 48.4 ± 24.4%, for mesenteric ischemia, 91.9 ± 4.8% for seizure, and 89.9 ± 3.5% for ‘others’.

**Fig. 5:**
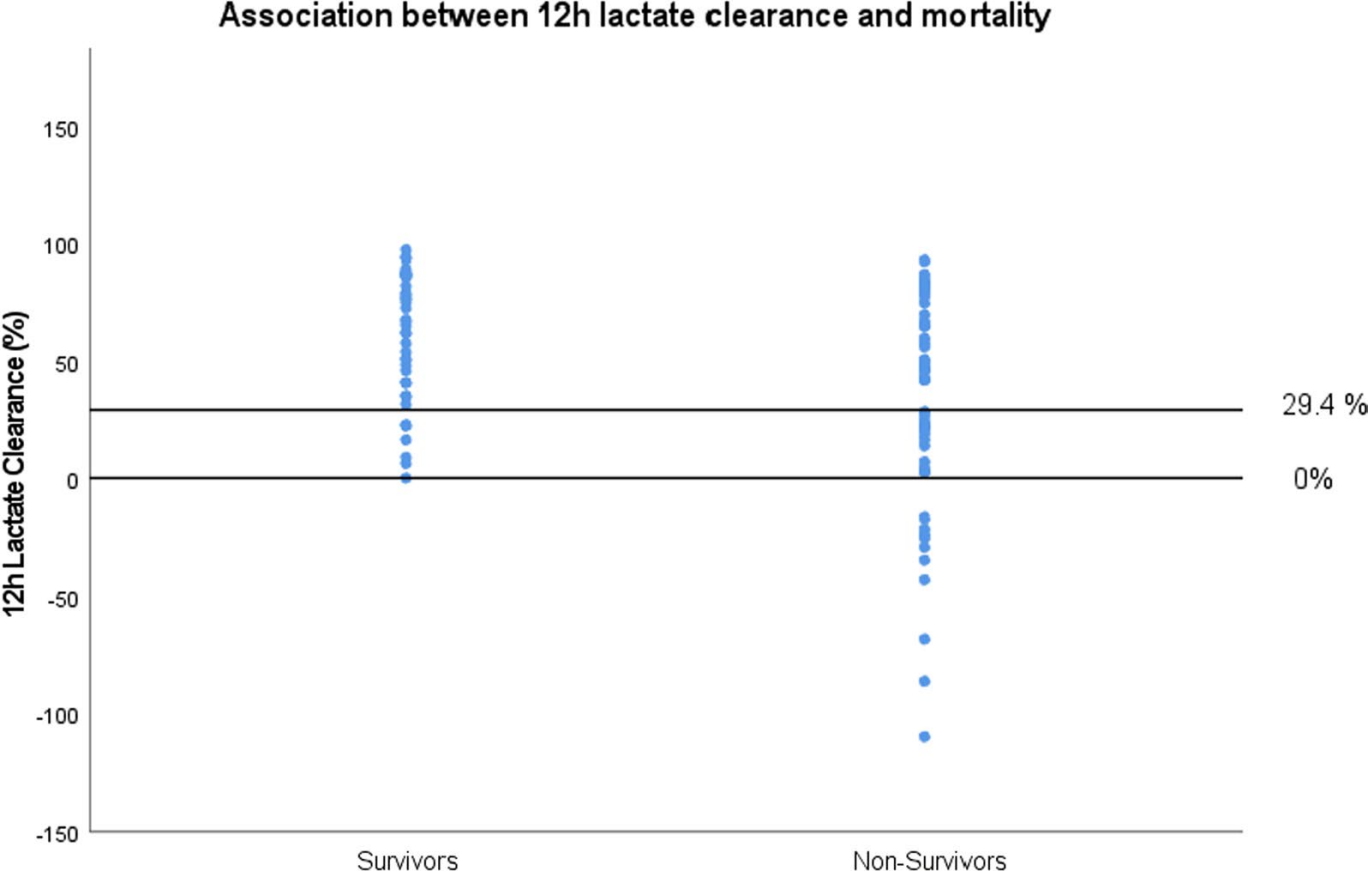
Association between 12 h lactate clearance and mortality. The *black lines* show 12 h lactate clearance of 0% and the cutoff having highest specificity to predict ICU mortality regarding sensitivity and specificity (12 h lactate clearance of 29.4%)

We summarized the association between 12 h lactate clearance and mortality in Fig. 5. Mortality rate for patients with negative lactate clearance (implying an increase of serum lactate level 12 h after first measurement of severe hyperlactatemia) was 100%.

## Discussion

This retrospective study indicates that severe hyperlactatemia is associated with poor clinical outcome in unselected surgical patients. Our results highlight that severe hyperlactatemia is still associated with an extremely high ICU mortality.

Studies of critically ill medical [12] and cardiac surgical [13, 14] showed an association between elevated lactate level, prolonged lactate clearance and mortality. Juneja et al. demonstrated that the outcome of patients with hyperlactatemia on admission is inferior, regardless of the occurrence of hypotension. Hyperlactatemia on admission can thus help us identify patients at higher risk of death so that immediate resuscitative measures can be taken to improve the overall survival.

We observed sepsis as the predominant reason for severe hyperlactatemia in our cohort. This is consistent with the findings of Haas et al. [11]. Gotmarker et al. have even demonstrated that patients with isolated hyperlactatemia in the setting of sepsis had significantly reduced 90-day overall survival compared to patients with isolated sepsis induced hypotension [15]. Various guidelines, including those by „surviving sepsis campaign’ [16] now assign lactate measurement as a screening strategy for identifying patients with suspected severe sepsis with higher risk of mortality.

Interestingly, our data suggest that patients whose hyperlactatemia results from sepsis show significantly better overall survival than patients with hyperlactatemia due to mesenteric ischemia (Fig. 4). Thus, it can be concluded that despite the apparent anaerobic processes occurring in both groups, the underlying etiology and reversibility of hyperlactatemia seem to be of relevance when evaluating overall survival. Mesenteric ischemia without hyperlactatemia is also associated with poor prognosis, which is also confirmed by other studies [17].

Furthermore, our results emphasize the importance of lactate clearance for the prognosis of overall survival. Patients with a lactate clearance of less than 30% within the first 12 h were shown to have a reduced overall survival with an ICU mortality of 80%. The reversibility of hyperlactatemia is therefore an important risk factor. Multiple trials [9–11, 18] showed a good predictive power for 12 h lactate clearance in determining ICU mortality in critical ill patients. Lactate clearance is currently not part of the general recommendations for septic shock management. Our data go in line with the findings of Arnold et al. [19] and show that assessment of lactate clearance is important as a predictor of mortality, independent of central venous oxygen saturation. We suggest that serial lactate measurement may provide important information during intensive care treatment and resuscitation.

Mak et al. [13] suggest that the duration of hyperlactatemia is a more important risk factor than the peak lactate concentration for cardiac surgical patients. The authors propose that persistent elevations in lactate are due to a state of ongoing reduced perfusion, leading to a significant increase in mortality risk. This is consistent with our findings regarding the correlation of reduced lactate clearance and increased mortality. Thus, an irreversible cause of hyperlactatemia and reduced hepatic and renal clearances due to multi-organ failure seem to be crucial in risk calculation. However, there are many studies for post-cardiac surgery patients regarding hyperlactatemia and lactate clearance but there is a need for prospective further studies evaluating general surgical patients.

Our findings confirm the association between the timing of severe hyperlactatemia and ICU mortality. Patients, who already showed severe hyperlactatemia at ICU admission, had a significantly increased risk of mortality comparing to patients with lactate levels < 10 mmol/L. This goes along with data of a European prospective study in patients with liver cirrhosis showing the significant impact of admission lactate levels on 28-day mortality [20]. Juneja et al. demonstrated that hyperlactatemia is commonly evident on admission to a general medical ICU and is associated with increased need for organ support and increased ICU mortality [12]. The outcome of patients with hyperlactatemia on admission is worse, independent of the presence of hypotension. Hyperlactatemia on admission can guide in identifying patients at higher risk of mortality [12].

Regarding our results, it can be assumed that patients with severe hyperlactatemia on admission have basically not received causal therapy so far, and thus have a worse outcome than patients who develop hyperlactatemia in the ICU receiving differentiated therapy, e.g., in the context of postoperative hemorrhage or seizure. This could explain the lower overall survival and is an important finding with regard to further prognosis-adapted therapy.

Our single-center retrospective study demonstrated that severe hyperlactatemia is associated with poor clinical outcome in unselected surgical patients. Moreover, we showed that a slow or negative lactate clearance is associated to higher mortality rates.

Our study focused on data resulting from surgical patients. In contrast to the study for Haas et al. we identified sepsis as the major cause of severe hyperlactatemia (51.9%). Cardiopulmonary resuscitation and cariogenic shock we identified as cause of severe hyperlactatemia only in a minority of our patients (5% and 2.5% respectively in contrast to 13.8% and 19.3% respectively showed in the study of Haas et al.). Based on our data, it would be important to perform further analysis regarding pathogenesis of hyperlactatemia in patients with sepsis and septic shock.

Interestingly and also important for the clinical praxis is the fact that patients whose hyperlactatemia resulted from mesenteric ischemia showed significantly lower overall survival than patients with hyperlactatemia due to sepsis. Although the cause of hyperlactatemia in this group of patients is often reversible (surgical resection of the infarcted gut combined with angioplasty to optimize the perfusion) it is to be highlighted, that lactate levels > 10 mmol/L were associated with poor outcomes. Especially, in this subgroup of surgical patients lactate levels should be not only closely monitored but also used as a major marker to guide the therapeutic approach.

To sum up, we believe that our study presented some interesting and valuable data regarding hyperlactatemia in surgical patients. From our clinical experience, the collective of these patients forms a high-risk group, which should be especially put in the focus of intensive care. While the data situation for cardiac surgical and medical patients is very good, the study of a general surgical collective, as in our case, is special.

Lactate is strong predictor of organ failure and short-term overall survival in critically ill surgical patients. Due to the simplicity and availability in the clinical ICU setting, lactate measurements should be used as a useful and rapid tool to assess severity of disease.

Limitations to this study are the retrospective nature as well as selection bias. Due to retrospective design time trends, selection bias and confounder due to incomplete data cannot be avoided. Furthermore, the lack of absolute diagnostic criteria for subgroup selection can lead to over- and/or underrepresentation of subgroups. Multiple factors can lead to severe hyperlactatemia, thus making it difficult to identify a sole cause of increased lactate levels. Nevertheless, we were able to show interesting results for more than 250 patients agreeing with previously reported observations: It is well-known that hyperlactatemia is associated with an increased risk of death [21–23]. Almost 30 years ago, Bakker et al. reported about 48 patients suffering from septic shock, with survivors showing significantly lower levels of lactate initially and in the final phase of septic shock, compared to the control group [21]. Another retrospective study of Varpula et al. with patients in septic shock revealed similar results emphasizing the impact of lactate on mortality [23]. A few years later, Khosravani et al. confirmed the results by reporting data of more than 10 000 ICU patients. Hyperlactatemia was commonly seen in critically ill patients and predicted the risk of death [22].

## Conclusion

Hyperlactatemia remains a life-threatening emergency status with a high mortality rate. The underlying etiology of hyperlactatemia is relevant to overall survival. Our findings match with previous studies indicating that lactate clearance predicts mortality. Nevertheless, the results clearly indicate the need for further prospective research to develop therapeutic approaches for a better management of hyperlactatemia dependent on its etiology and consequently improve outcome of affected patients.

## Data Availability

The original anonymous dataset is available on request from the corresponding author at julia.spiegelberg@uniklinik-freiburg.de.

## Abbreviations

AUC: Area under the curve
ROC: Receiver operating characteristic
SD: Standard deviation
ANOVA: Analysis of variance
ARDS: Acute respiratory distress syndrome
ICU: Intensive care unit
